# The proto-oncogene KRAS is targeted by miR-200c

**DOI:** 10.18632/oncotarget.1427

**Published:** 2013-11-24

**Authors:** Florian Kopp, Ernst Wagner, Andreas Roidl

**Affiliations:** ^1^ Pharmaceutical Biotechnology, Department of Pharmacy, Ludwig-Maximilians-Universität München, Munich, Germany

**Keywords:** breast cancer, lung cancer, miRNA, K-ras, cell cycle, proliferation

## Abstract

The GTPase K-ras is involved in a variety of cellular processes such as differentiation, proliferation and survival. However, activating mutations, which frequently occur in many types of cancer, turn KRAS into one of the most prominent oncogenes. Likewise, miR-200c is a key player in tumorigenesis functioning as a molecular switch between an epithelial, non-migratory, chemosensitive and a mesenchymal, migratory, chemoresistant state. While it has been reported that KRAS is modulated by several tumor suppressor miRNAs, this is the first report on the regulation of KRAS by miR-200c, both playing a pivotal role in oncogenesis. We show that KRAS is a predicted target of miR-200c and that the protein expression of KRAS inversely correlates with the miR-200c expression in a panel of human breast cancer cell lines. KRAS was experimentally validated as a target of miR-200c by Western blot analyses and luciferase reporter assays. Furthermore, the inhibitory rffect of miR-200c-dependent KRAS silencing on proliferation and cell cycle was demonstrated in dfferent breast and lung cancer cell lines. Thereby, the particular role of KRAS was dissected from the role of all the other miR-200c targets by specific knockdown experiments using siRNA against KRAS. Cell lines harboring an activating KRAS mutation were similarly affected by miR-200c as well as by the siRNA against KRAS. However, in a cell line with wild-type KRAS only miR-200c was able to change proliferation and cell cycle. Our findings suggest that miR-200c is a potent inhibitor of tumor progression and therapy resistance, by regulating a multitude of oncogenic pathways including the RAS pathway. Thus, miR-200c may cause stronger anti-tumor efffects than a specific siRNA against KRAS, emphasizing the potential role of miR-200c as tumor suppressive miRNA

## INTRODUCTION

In cancer, many cellular processes such as differentiation, growth, migration and survival, are regulated by GTPases of the RAS family. Amongst them K-ras plays a pivotal role in oncogenesis due to its capability of transforming human cells into malignant tumor cells particularly when harboring an activating mutation in codon 12 or 13. *KRAS* mutations frequently occur in many types of human tumors, for example 70 – 90% in pancreas, 30 – 60% in colon and 15 – 50% in lung, making *KRAS* one of the most prominent oncogenes [1, 2]. Furthermore, activating oncogenic *KRAS* mutations are often associated with resistance to chemotherapy and targeted therapies [2-6]. Due to the poor prognosis for cancer patients with mutated *KRAS*, much effort has been spent on developing specific therapies for targeting oncogenic *KRAS*. However, apart from specific RNAi methods, up to now there are no small molecules available which can specifically target K-ras.

miRNAs are endogenous regulators of *KRAS* and many other cellular pathways. Their differential expression in various cancerous tissues compared to normal tissues influences tumorigenesis [7], turning them either into tumor suppressors or oncomirs [8, 9]. It has been shown that the let-7 family inhibits *KRAS* [10] resulting in slower proliferation and tumor growth of lung cancer cells [11-13]. Moreover, miR-143 has been demonstrated to regulate tumorigenesis in colorectal and prostate cancer cells by targeting *KRAS* [14, 15]. In pancreatic carcinogenesis it has been reported that the oncogene *EVI1* leads to the activation of the *KRAS* pathway through suppression of the *KRAS* suppressor miR-96 [16]. A recent study has revealed that miR-30c targets the *KRAS* oncogene as well and is deregulated in hereditary breast cancer [17]. In contrast to these tumor suppressor miRNAs, which generally display a low expression level in cancer cells, miR-200c is differentially expressed among cancer cells and acts as important molecular switch by modulating a multitude of cellular processes. miR-200c regulates epithelial-mesenchymal transition (EMT) by inhibiting *ZEB1* and *ZEB2*, the transcriptional repressors of the epithelial marker E-cadherin [18-20]. By the inhibition of EMT and the regulation of several other genes important for cell motility, miR-200c reduces the migration and invasion of cancer cells, preventing tumor dissemination and metastases [21-25]. It has also been shown that miR-200c links breast cancer stem cells with normal stem cells and that downregulation of miR-200 is required for the formation and maintenance of cancer stem cells [26, 27]. Moreover, in resistant cancer cells miR-200c is able to restore sensitivity to anti-EGFR therapy [28, 29] and to classical chemotherapeutics such as paclitaxel or doxorubicin [30-33]. Therefore, miR-200c targeting *KRAS* is of great interest in order to understand and predict tumor progression and therapy susceptibility of cancer patients.

Here, we report that *KRAS* is targeted by miR-200c, which results in a slower proliferation and in an altered cell cycle of cancer cells. The alterations are dependent on the presence of *KRAS* mutations and occur in different types of cancer.

## RESULTS

### *KRAS* is a predicted target of miR-200c and its protein expression inversely correlates with miR-200c expression in breast cancer cells

**Figure 1:**
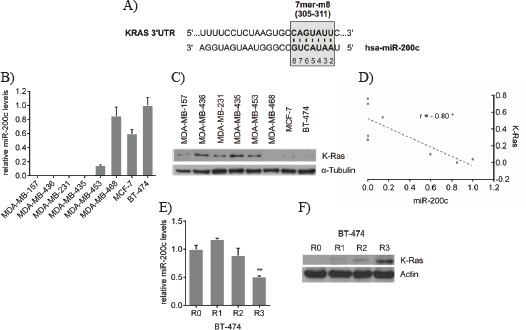
*KRAS* is a predicted target of miR-200c and its protein expression inversely correlates with miR-200c expression in breast cancer cells A) Target site prediction of miR-200c in the 3'UTR of the *KRAS* gene. By means of the three different prediction algorithms TargetScan, miRanda and DIANA microT, a unique conserved binding site with a 7mer-m8 seed at position 305 – 311 of the 3'UTR of the *KRAS* gene was found. B) miR-200c expression in a panel of breast cancer cell lines. miR-200c expression was normalized to miR-191 and values are stated as mean ± SD (n=3). C) K-ras protein expression in a panel of breast cancer cell lines. Total cell lysates were subjected to Western blot analysis and incubated with indicated antibodies. D) Correlation of K-ras protein and miR-200c expression. The values of the relative K-ras protein and miR-200c expression are listed in Table 1. The graph shows the Pearson correlation scatter plot of the relative K-ras and miR-200c levels in the different breast cancer cell lines (*p<0.05). Chemotherapeutic treatment of the miR-200c^high^ cell line BT-474. Cells were sequentially treated with 50nM doxorubicin. After each cycle cells were harvested for RNA isolation and protein lysates to determine E) the relative miR-200c expression and F) the K-ras protein levels of the indicated round. Values are stated as mean ± SD (n=3). For statistical analysis a student's t-test was performed (**p<0.01; R0 compared to R3).

In order to examine whether miR-200c has a putative target site in the 3'UTR of the *KRAS* gene, online prediction tools were utilized, which were based on the three different algorithms TargetScan [34], miRanda [35] and DIANA microT [36, 37]. All applied algorithms uniformly predicted one specific binding site, which is broadly conserved among several species. This predicted site is located at position 305 – 311 of the *KRAS* 3'UTR and comprises a 7mer-m8 seed, i.e. a perfect base pairing between the nucleotides 2 – 7 (seed region) and the nucleotide 8 of the mature miRNA and its target mRNA (Figure 1A). As miR-200c is well established and known to be differentially expressed in breast tumors, miR-200c (Figure 1B) and K-ras protein (Figure 1C) expression levels were analyzed in a panel of different breast cancer cell lines (a numerical table is given in Table 1). The expression of miR-200c was found to inversely correlate with the K-ras protein expression (Figure 1D); i.e. breast cancer cells, which displayed a high miR-200c expression, had low protein levels of K-ras (Pearson r = -0.80). A typical characteristic of advanced cancer is acquired chemoresistance. Since miR-200c as well as particularly mutated K-ras have both been implicated in resistance to classical chemotherapy and targeted therapies [4, 5, 28-33], we investigated whether a reduction of miR-200c increases K-ras protein expression in an assay for induced chemoresistance [32]. The miR-200c-positive breast cancer cell line BT-474 was treated with doxorubicin for three rounds, which resulted in reduced miR-200c (Figure 1E) and elevated K-ras protein levels (Figure 1F) suggesting a miR-200c-dependent regulation of *KRAS*.

**Table 1:** Relative miR-200c and K-ras protein expression in the panel of breast cancer cell lines

Cell line	Relative miR-200c expression [fold]	Relative K-ras protein expression [ratio]
MDA-MB-157	0.001	0.27
MDA-MB-436	0.001	0.70
MDA-MB-231	0.002	0.32
MDA-MB-435	0.000	0.76
MDA-MB-453	0.142	0.54
MDA-MB-468	0.850	0.00
MCF-7	0.597	0.10
BT-474	1.000	0.04

The values of the relative miR-200c expression from Figure 1B were stated as fold expression of BT-474 cells. The relative intensities of the Western blot from Figure 1C were quantified using ImageJ software. K-ras signals were then normalized to the loading control α-tubulin and depicted as ratio.

### miR-200c inhibits K-ras protein expression without affecting *KRAS* mRNA levels

**Figure 2:**
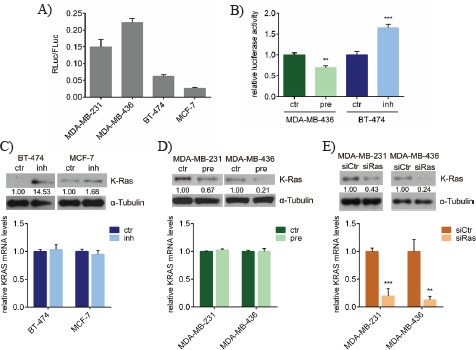
miR-200c inhibits K-ras protein expression without affecting *KRAS* mRNA levels A) Luciferase reporter assay with different breast cancer cell lines. The renilla luciferase reporter containing the 3'UTR of *KRAS* including the predicted target site of miR-200c (RLuc) or the firefy luciferase control plasmid pGL3 (FLuc) were transfected into the indicated cell lines. Renilla reporter luciferase activity was normalized to the activity of the firefy control as ratio (RLuc/FLuc). Values are stated as mean ± SEM (n=5). B) Luciferase reporter assay upon miR-200c modulation. MDA-MB-436 cells were transfected with either pre-miR-200c (pre) or scrambled pre-miR-control (ctr). BT-474 cells were transfected with either miR-200c inhibitor (inh) or scrambled control inhibitor (ctr). Relative luciferase activities (RLuc/FLuc) are depicted in the graph. Values are stated as mean ± SEM (n=5). For statistical analysis a student's t-test was performed (**p<0.01; ***p<0.001). C) miR-200c inhibition in the miR-200c^high^ cell lines BT-474 and MCF-7. Indicated cell lines were transfected with either miR-200c inhibitor (inh) or scrambled control inhibitor (ctr) and at 72h post transfection subjected to Western blot analysis (upper panel) or quantitative RT-PCR (lower panel). Values are stated as mean ± SD (n=3). D) miR-200c overexpression in the miR-200c^low^ cell lines MDA-MB-231 and MDA-MB-436. Indicated cell lines were transfected with either pre-miR-200c (pre) or scrambled pre-miR-control (ctr) and at 72h after transfection subjected to Western blot analysis (upper panel) or quantitative RT-PCR (lower panel). Values are stated as mean ± SD (n=3). E) *KRAS*-specific knockdown in MDA-MB-231 and MDA-MB-436. Cells were transfected either with a siRNA pool against human *KRAS* (siRas) or with a non-targeting control siRNA (siCtr). After 72h K-ras protein (upper panel) and *KRAS* mRNA levels (lower panel) were determined. Values are stated as mean ± SD (n=3). For statistical analysis a student's t-test was performed (**p<0.01; ***p<0.001).

For the validation of *KRAS* as a novel target of miR-200c, a luciferase reporter assay was performed using a vector encoding for renilla luciferase and almost the entire 3'UTR of the *KRAS* gene including the predicted miR- 200c binding site. Ectopic expression of this reporter in two miR-200c^low^ (MDA-MB-231 and MDA-MB-436) and two miR-200c^high^ (BT-474 and MCF-7) breast cancer cell lines showed high and low luciferase activities, respectively (Figure 2A). This correlation indicates a direct inhibition of the luciferase reporter via the *KRAS* 3'UTR by miR-200c. Next, it was examined whether miR-200c was able to regulate the luciferase reporter when overexpressed or inhibited. The luciferase reporter was therefore transfected together with pre-miR-200c in MDA-MB-436 cells or miR-200c inhibitor in BT-474 cells. As expected, overexpression of miR-200c led to a decreased luciferase activity, whereas its inhibition resulted in an enhanced bioluminescence (Figure 2B). To further prove the inhibition of *KRAS* expression by miR-200c, protein and mRNA levels were determined after either miR-200c inhibition or overexpression. Inhibition of miR-200c in BT-474 and MCF-7 cells led to an elevated K-ras protein expression, while *KRAS* mRNA levels were not changed (Figure 2C). On the other hand, overexpression of miR-200c resulted in decreased K-ras protein and unaltered *KRAS* mRNA levels in MDA-MB-231 and MDA-MB-436 cells (Figure 2D). Moreover, in the murine breast cancer cell line 4T1, which endogenously displayed a medium expression of miR-200c (Supplement Figure S1), it was demonstrated that K-ras protein levels were both down-and up-regulated after miR-200c overexpression and inhibition (Supplement Figure S2). In order to assess the silencing efficiency and the mechanism of the miR-200c-induced *KRAS* knockdown, the effects of miR-200c were compared with those of a siRNA-pool against *KRAS* (siRas). While the siRNA knockdown was similar on protein level, siRas remarkably reduced *KRAS* mRNA (Figure 2E), consistent with the different modes of action of miRNA- and siRNA-induced gene silencing. Although it has been reported that miRNAs can also down-regulate target mRNAs by affecting their stabilities [38], miR-200c primarily inhibits the translation of *KRAS*, whereas siRas causes the expected mRNA cleavage.

### *KRAS* silencing by miR-200c and siRas leads to reduced proliferation and changed cell cycle of breast cancer cells dependent on the *KRAS* mutation status

**Figure 3:**
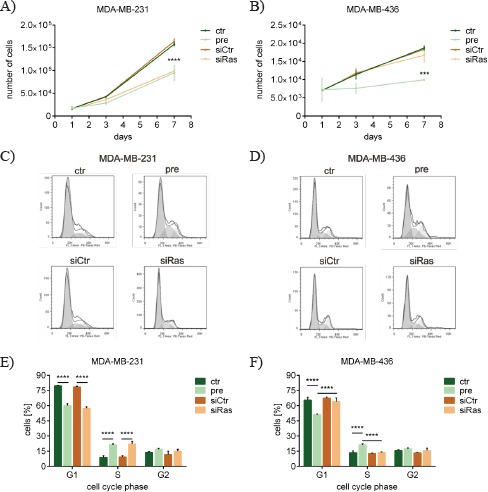
*KRAS* silencing by miR-200c and siRas leads to reduced proliferation and changed cell cycle of breast cancer cells dependent on the *KRAS* mutation status Proliferation of different breast cancer cell lines upon *KRAS* knockdown. A) MDA-MB-231 cells which harbor an activating (G13D) *KRAS* mutation and B) MDA-MB-436 cells which express wild-type *KRAS* were transfected with pre-miR-200c (pre), scrambled pre-miR-control (ctr), siRNA against *KRAS* (siRas) or non-targeting control siRNA (siCtr). Values are stated as mean cell number ± SD (n=3). For statistical analysis a student's t-test was performed (***p<0.001; ****p<0.0001). Cell cycle of different breast cancer cell lines upon *KRAS* knockdown. C) MDA-MB-231 and D) MDA-MB-436 cells were subjected to flowcytometry at 72h after transfection with the indicated oligonucleotides. Cell cycle analysis was carried out using FlowJo software. Results are presented as histograms (y-axis: counts; x-axis: PE-Texas Red indicative for propidium iodide). Statistical analysis of the cell cycle phases upon *KRAS* silencing. The percentage of E) MDA-MB-231 and F) MDA-MB-436 cells in the respective cell cycle phase upon indicated oligonucleotide transfection was determined. Values are stated as mean ± SD (n=3). For statistical analysis a student's t-test was performed (****p<0.0001)

The significance of the oncogene *KRAS* is underlined by frequently occurring activating mutations in numerous tumors and cancer cell lines. According to the respective mutation status, different physiological effects were expected upon *KRAS* knockdown. Thus, the cell line MDA-MB-231, which harbors an activating G13D mutation in the *KRAS* gene, and the cell line MDA-MB-436, which expresses the wild-type *KRAS* gene [39, 40], were chosen for further experiments. By using pre-miR-200c as well as siRas for the silencing of *KRAS*, the particular role of *KRAS* should be dissected from the role of all the other targets of miR-200c. Several reports have shown that oncogenic K-ras stimulates proliferation in various cell types, highlighting its role in tumorigenesis [41-43]. Therefore, the proliferation of the two breast cancer cell lines was analyzed upon transfection with pre-miR-200c or siRas. In the *KRAS* mutated cell line MDA-MB-231 the proliferation of pre-miR-200c- and siRas-transfected cells was similarly decreased (Figure 3A), whereas in the *KRAS* wild-type cell line MDA-MB-436 only pre-miR-200c was able to significantly reduce proliferation (Figure 3B). As it has been demonstrated that oncogenic K-ras drives cell cycle progression by enabling cells to enter the S-phase and thereby promotes tumorigenesis [44, 45], cell cycle analyses were additionally performed to investigate whether the cell cycle was differentially affected. Consistent with the proliferation, the cell cycle of MDA-MB-231 cells was considerably changed upon both pre-miR-200c and siRas transfection (Figure 3C), whereas only pre-miR-200c changed the cell cycle of MDA-MB-436 cells (Figure 3D). Quantification of the cell cycle phases revealed that in MDA-MB-231 cells pre-miR-200c as well as siRas led to a decrease of the G1-phase and an increase of the S-phase (Figure 3E). In MDA-MB-436 cells, however, only pre-miR-200c achieved a reduction of the G1-phase and an increase of the S-phase, whereas siRas did not affect the cell cycle (Figure 3F).

### miR-200c and siRas also affect the cell cycle of lung cancer cells by inhibiting *KRAS*

As the relevance of *KRAS* mutations in breast cancer remains elusive, the physiological effects of miR-200c-dependent *KRAS* silencing were explored in a more relevant cancer type. Besides in pancreas and colon cancer, *KRAS* mutations occur very frequently in non-small cell lung cancer (NSCLC) (15-50%) [2, 4].

**Figure 4:**
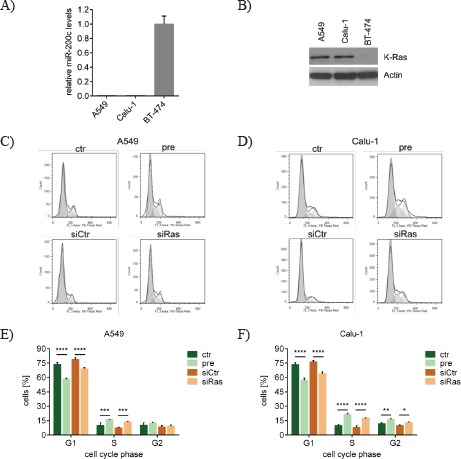
miR-200c and siRas also affect the cell cycle of lung cancer cells by inhibiting *KRAS* A) miR-200c expression and B) K-ras protein levels of the two *KRAS* mutated lung cancer cell lines A549 (G12S) and Calu-1 (G12D) in comparison to the breast cancer cell line BT-474. Values are stated as mean ± SD (n=3). Cell cycle of different lung cancer cell lines upon *KRAS* knockdown. C) A549 cells and D) Calu-1 cells were subjected to flowcytometry at 72h after transfection with pre-miR-200c (pre), scrambled pre-miR-control (ctr), siRNA against *KRAS* (siRas) or non-targeting control siRNA (siCtr). Cell cycle analysis was carried out using FlowJo software. Results are presented as histograms (y-axis: counts; x-axis: PE-Texas Red indicative for propidium iodide). Statistical analysis of the cell cycle phases upon *KRAS* silencing. The percentage of E) A549 and F) Calu-1 cells in the respective cell cycle phase upon indicated oligonucleotide transfection was analyzed. Values are stated as mean ± SD (n=3). For statistical analysis a student's t-test was performed (*p<0.05; **p<0.01; ***p<0.001; ****p<0.0001).

Thus, the two NSCLC cell lines A549 and Calu-1 were used, which harbor the activating *KRAS* mutations G12S and G12C, respectively [46, 47]. Both lung cancer cell lines displayed low miR-200c levels (Figure 4A) but a considerable K-ras protein expression (Figure 4B) as compared to the miR-200c^high^ breast cancer cell line BT-474. The cell cycle upon *KRAS* knockdown was determined in the two lung cancer cell lines to examine whether the effects were comparable with those of the *KRAS* mutated breast cancer cell line MDA-MB-231. Of note, the cell cycle of pre-miR-200c- as well as siRas-transfected A549 (Figure 4C) and Calu-1 (Figure 4D) cells was similarly changed. In accordance with MDA-MB-231 cells, the G1-phase was decreased, whereas the S-phase was increased in A549 (Figure 4E) and Calu-1 (Figure 4F) cells. These data suggest that miR-200c can generally interfere with cell proliferation and cell cycle by directly targeting oncogenic *KRAS* independent of the respective cancer type. Furthermore, these results highlight the prominent role of the miR-200c-dependent regulation of *KRAS*, especially when applied to cell lines which are driven by oncogenic *KRAS*.

## DISCUSSION

Various cellular processes, such as differentiation, proliferation and survival are regulated by the GTPase K-ras. Frequently occurring activating mutations turn *KRAS* into one of the most prominent oncogenes. Similarly, a pivotal role in tumorigenesis as a molecular switch between an epithelial, non-migratory, chemosensitive and a mesenchymal, migratory, chemoresistant state has been attributed to miR-200c. While it has been reported that *KRAS* is regulated by several tumor suppressor miRNAs, this is the first report on the direct regulation of *KRAS* by miR-200c.

Thereby, *KRAS* was experimentally validated as a target of miR-200c by Western blot analyses and luciferase reporter assays. Interestingly, upon molecular evolution, an assay for acquired chemoresistance in which cancer cells were sequentially treated with doxorubicin, BT-474 cells displayed a significantly reduced miR-200c and a remarkably enhanced K-ras protein expression. Even though BT-474 cells express wild-type K-ras [39], the up-regulation of K-ras is a reasonable way to overcome the chemotherapeutic treatment as those cells have an amplification of the pro-survival gene *ERBB2* encoding for the receptor tyrosine kinase Her2 [48], which signals downstream among others via the RAS/MAPK signaling pathway. This suggests that not only the presence of mutated but also the expression levels of wild-type K-ras are important for tumor progression and may serve as predictive marker for therapy efficacy, especially when occurring in combination with other genetic alterations such as *EGFR* mutations or *ERBB2* amplifications.

Furthermore, in different breast and lung cancer cell lines an inhibitory effect of miR-200c-dependent *KRAS* silencing on proliferation and cell cycle was demonstrated. Specific knockdown experiments using siRNA against *KRAS* dissected the particular role of *KRAS* from the role of the other miR-200c targets. miR-200c as well as the siRNA against *KRAS* similarly affected the cell cycle of MDA-MB-231, A549 and Calu-1 cells, which harbor an activating *KRAS* mutation. However, in the *KRAS* wild-type cell line MDA-MB-436 only miR-200c was able to change proliferation and cell cycle. Uhlmann *et al*. [49] have shown that in MDA-MB-231 cells the miR-200bc/429 seed-cluster, in particular miR-200c, inhibits EGF-driven invasion as well as proliferation and cell cycle progression, the latter by decreasing the G1-population. However, they ascribed the physiological effects to a miR-200c-induced down-regulation of PLC gamma 1, regardless of the *KRAS* mutation status. While the effects on invasion were nicely refected by a specific knockdown of PLC gamma 1, the effects on proliferation were only mimicked in part. Here, it was shown that the effects on proliferation and cell cycle are coinciding for miR-200c and siRas if an activating *KRAS* mutation is present. These results indicate that in MDA-MB-231 cells, which harbor the activating *KRAS* mutation G13D, miR-200c inhibits proliferation and cell cycle progression more likely via a down-regulation of *KRAS*. On the contrary, in cells harboring wild-type *KRAS*, only miR-200c is able to alter proliferation and cell cycle presumably via other targets than *KRAS*, for instance *BMI1*. The polycomb group repressor Bmi1 induces transcriptional repression of a variety of genes including p16^Ink4a^ of the Ink4a locus, which causes cell cycle arrest and senescence [50]. Hence, miR-200c shows a broader efficacy than siRas against cancer cells by targeting multiple genes and pathways.

**Figure 5:**
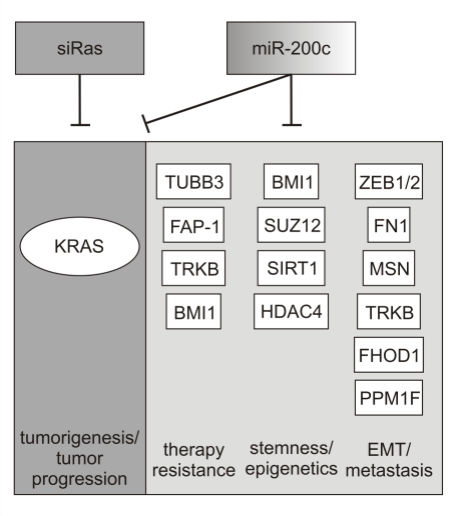
Regulatory network of miR-200c While siRas can only specifically target *KRAS* mRNA, miR-200c regulates a variety of genes involved in tumor progression, metastasis and therapy resistance. By controlling a multitude of cellular processes, miR-200c causes stronger effects against various cancer cells independent of the mutational status of *KRAS*. The targets depicted in the diagram and their respective biological roles are reviewed in [53].

Activating mutations in the *KRAS* gene are important drivers of carcinogenesis in many types of cancer, such as lung, colon and pancreas [1, 2]. However, it has been reported that human tumors display a remarkable intratumoral heterogeneity [51], which is furthermore associated with drug resistance and the failure of cancer therapies [52]. Oncogenic K-ras might not necessarily be expressed across an entire tumor, but rather cellular subpopulations may exist which display the wild-type *KRAS* gene. These cell populations can additionally contribute to tumor progression and to an aggressive phenotype including a high propensity of cancer cells to metastasize and to overcome drug treatment. Therefore, usage of miR-200c in a therapeutic approach may be superior to that of a siRNA against *KRAS*. As demonstrated by several reports, this miRNA has multiple targets [53], which regulate crucial events for tumor progression, e.g. epithelial-mesenchymal transition (EMT), acquisition of stem-like properties or therapy resistance (Figure 5).

Moreover, KEGG-pathway analysis of all potential miR-200c targets predicted by TargetScan [34] revealed that miR-200c might strongly interact with the MAPK and ERBB signaling pathway by regulating a multitude of target genes, such as central adaptor proteins like Shc and Sos, kinases like *MEKK1* and *PKC* or transcription factors like *SRF* and *JUN* (Supplement Figure S3). Although the direct regulation of these targets needs to be proven, these findings indicate that miR-200c may have additional regulating functions as a kind of gatekeeper of tumor progression and therapy resistance by controlling a complex network of oncogenic pathways including the RAS/MAPK pathway.

## METHODS

### Oligonucleotides

The following oligonucleotides were used: miRCURY LNA miRNA Inhibitor for hsa-miR-200c (inh) (Exiqon), miRCURY LNA miRNA Inhibitor Control Negative Control A (ctr) (Exiqon), Pre-miR miRNA Precursor of hsa-miR-200c (pre) (Ambion), Pre-miR miRNA Negative Control (ctr) (Ambion), ON-TARGETplus SMARTpool siRNA against human *KRAS* consisting of four different siRNAs (Dharmacon) (siRas) and the non-targeting control siRNA (siCtr), which was previously described [54].

### Cell Culture

The breast cancer cell lines MDA-MB-157, MDA-MB-436, MDA-MB-231, MDA-MB-435 (formerly regarded as breast cancer, in fact melanoma), MDA-MB-453, MDA-MB-468, MCF-7, BT-474 and 4T1 as well as the lung cancer cell lines A549 and Calu-1 were cultivated according to supplier's instructions (ATCC).

### Quantitative RT-PCR

Total RNA was isolated followed by a reverse transcription and a quantitative RT-PCR as described previously [32]. Primers and UPL hydrolysis probes (Roche) are listed in Supplement Table S1.

### Cell Lysis and Immunoblotting

Western blot experiments were performed as previously described [32] using the primary antibodies against the following proteins: K-ras (415700, Invitrogen), actin (I-19) (SC-1616, Santa Cruz) and α-tubulin (DM-1A) (T9026, Sigma). Western blots were quantified using ImageJ software [55]. The K-ras intensities were thereby normalized to the intensities of the loading control (actin or α-tubulin) and presented as ratio or fold expression.

### Doxorubicin Treatment of BT-474 Cells

BT-474 cells were sequentially treated with 50nM doxorubicin (doxorubicin hydrochloride, Sigma) for 72h as described previously [32]. As soon as the cells recovered (confluence of 80%) the next treatment round was started. After each cycle cells were harvested for RNA isolation and protein lysates.

### Oligonucleotide Transfections

Indicated cell lines were transfected with the above mentioned oligonucleotides using Lipofectamine 2000 (Invitrogen) according to manufacturer's protocol. The following nucleotide amounts were thereby used: in 6-well plates (used for quantitative RT-PCR and Western blot) 75pmol, in 24-well plates (used for cell cycle) 15pmol, in 48-well plates (used for proliferation) 7.5pmol and in 96-well plates (used for luciferase assays) 3pmol.

### Luciferase Reporter Assays

A renilla luciferase reporter plasmid containing the almost entire 3'UTR of the *KRAS* gene was obtained from Addgene (Addgene plasmid 44571). This plasmid was generated by Frank Slack and is based on the plasmid with the let-7 mutated binding site LCS6m from the publication Chin *et al*. [56]. The renilla luciferase reporter containing the 3'UTR of *KRAS* (RLuc) or the firefly luciferase control plasmid pGL3 (Promega) (FLuc) were transfected into the indicated cell lines using Lipofectamine 2000 (Invitrogen) according to manufacturer's instructions. All RLuc signals were normalized to the respective FLuc signals as ratio (RLuc/FLuc). Additionally, MDA-MB-436 cells were transfected with either pre-miR-200c (pre) or pre-miR-control (ctr) (Ambion) together with the reporter plasmid RLuc and the control plasmid FLuc for normalization. BT-474 cells were transfected with either the LNA miR-200c inhibitor (inh) or the LNA control inhibitor (ctr) (Exiqon) together with the reporter plasmid RLuc and the control plasmid FLuc for normalization. All luciferase assays were performed in 96-well plates at 48h post transfection.

### Proliferation Assay

To examine proliferation, cells were seeded in 48-well plates. The following day, cells were transfected with indicated oligonucleotides and starting with day one after transfection proliferation was measured over seven days at indicated time points using a Scepter cell counter (Millipore).

### Cell Cycle Analysis

For the acquisition of the cell cycle, cells were seeded in 24-well plates and transfected with the indicated oligonucleotides the following day. At 72h after transfection the cells were subjected to flowcytometry. For this purpose, cells were trypsinized, centrifuged and incubated on ice for four hours in propidium iodide staining solution consisting of 0.1% sodium citrate, 0.1% Triton X-100 and 50µg/ml propidium iodide. Thereafter, cells were centrifuged, resuspended in phosphate buffered saline and analyzed with a CyAn ADP flowcytometer (Beckman Coulter). Doublets were discriminated by gating forward versus sideward scatter and forward scatter versus pulse width. The DNA content was measured through excitation of the dye at 488nm and detection of the emission with a 613/20 bandpass filter. Cell cycle analysis was carried out using FlowJo software.

The authors declare no conflict of interest.

## Supplementary Figures and Tables





## References

[1] Campbell PM, Der CJ (2004). Oncogenic Ras and its role in tumor cell invasion and metastasis. Seminars in cancer biology.

[2] Friday BB, Adjei AA (2005). K-ras as a target for cancer therapy. Biochimica et biophysica acta.

[3] Califano R, Landi L, Cappuzzo F (2012). Prognostic and predictive value of K-RAS mutations in non-small cell lung cancer. Drugs.

[4] Vakiani E, Solit DB (2011). KRAS and BRAF: drug targets and predictive biomarkers. The Journal of pathology.

[5] Wheeler DL, Dunn EF, Harari PM (2010). Understanding resistance to EGFR inhibitors-impact on future treatment strategies. Nature reviews Clinical oncology.

[6] Riely GJ, Marks J, Pao W (2009). KRAS mutations in non-small cell lung cancer. Proceedings of the American Thoracic Society.

[7] Calin GA, Croce CM (2006). MicroRNA signatures in human cancers.

[8] Esquela-Kerscher A, Slack FJ (2006). Oncomirs - microRNAs with a role in cancer. Nature reviews Cancer.

[9] Kent OA, Mendell JT (2006). A small piece in the cancer puzzle: microRNAs as tumor suppressors and oncogenes. Oncogene.

[10] Johnson SM, Grosshans H, Shingara J, Byrom M, Jarvis R, Cheng A, Labourier E, Reinert KL, Brown D, Slack FJ (2005). RAS is regulated by the let-7 microRNA family. Cell.

[11] Esquela-Kerscher A, Trang P, Wiggins JF, Patrawala L, Cheng A, Ford L, Weidhaas JB, Brown D, Bader AG, Slack FJ (2008). The let-7 microRNA reduces tumor growth in mouse models of lung cancer. Cell cycle.

[12] Johnson CD, Esquela-Kerscher A, Stefani G, Byrom M, Kelnar K, Ovcharenko D, Wilson M, Wang X, Shelton J, Shingara J, Chin L, Brown D, Slack FJ (2007). The let-7 microRNA represses cell proliferation pathways in human cells. Cancer research.

[13] Kumar MS, Erkeland SJ, Pester RE, Chen CY, Ebert MS, Sharp PA, Jacks T (2008). Suppression of non-small cell lung tumor development by the let-7 microRNA family. Proceedings of the National Academy of Sciences of the United States of America.

[14] Chen X, Guo X, Zhang H, Xiang Y, Chen J, Yin Y, Cai X, Wang K, Wang G, Ba Y, Zhu L, Wang J, Yang R, Zhang Y, Ren Z, Zen K (2009). Role of miR-143 targeting KRAS in colorectal tumorigenesis. Oncogene.

[15] Xu B, Niu X, Zhang X, Tao J, Wu D, Wang Z, Li P, Zhang W, Wu H, Feng N, Wang Z, Hua L, Wang X (2011). miR-143 decreases prostate cancer cells proliferation and migration and enhances their sensitivity to docetaxel through suppression of KRAS. Molecular and cellular biochemistry.

[16] To MD, Rosario RD, Westcott PM, Banta KL, Balmain A (2012). Interactions between wild-type and mutant Ras genes in lung and skin carcinogenesis. Oncogene.

[17] Tanic M, Yanowsky K, Rodriguez-Antona C, Andres R, Marquez-Rodas I, Osorio A, Benitez J, Martinez-Delgado B (2012). Deregulated miRNAs in hereditary breast cancer revealed a role for miR-30c in regulating KRAS oncogene. PloS one.

[18] Gregory PA, Bert AG, Paterson EL, Barry SC, Tsykin A, Farshid G, Vadas MA, Khew-Goodall Y, Goodall GJ (2008). The miR-200 family and miR-205 regulate epithelial to mesenchymal transition by targeting ZEB1 and SIP1. Nature cell biology.

[19] Hurteau GJ, Carlson JA, Spivack SD, Brock GJ (2007). Overexpression of the microRNA hsa-miR-200c leads to reduced expression of transcription factor 8 and increased expression of E-cadherin. Cancer research.

[20] Korpal M, Lee ES, Hu G, Kang Y (2008). The miR-200 family inhibits epithelial-mesenchymal transition and cancer cell migration by direct targeting of E-cadherin transcriptional repressors ZEB1 and ZEB2. The Journal of biological chemistry.

[21] Burk U, Schubert J, Wellner U, Schmalhofer O, Vincan E, Spaderna S, Brabletz T (2008). A reciprocal repression between ZEB1 and members of the miR-200 family promotes EMT and invasion in cancer cells. EMBO reports.

[22] Gibbons DL, Lin W, Creighton CJ, Rizvi ZH, Gregory PA, Goodall GJ, Thilaganathan N, Du L, Zhang Y, Pertsemlidis A, Kurie JM (2009). Contextual extracellular cues promote tumor cell EMT and metastasis by regulating miR-200 family expression. Genes & development.

[23] Howe EN, Cochrane DR, Richer JK (2011). Targets of miR-200c mediate suppression of cell motility and anoikis resistance. Breast cancer research : BCR.

[24] Jurmeister S, Baumann M, Balwierz A, Keklikoglou I, Ward A, Uhlmann S, Zhang JD, Wiemann S, Sahin O (2012). MicroRNA-200c represses migration and invasion of breast cancer cells by targeting actin-regulatory proteins FHOD1 and PPM1F. Molecular and cellular biology.

[25] Olson P, Lu J, Zhang H, Shai A, Chun MG, Wang Y, Libutti SK, Nakakura EK, Golub TR, Hanahan D (2009). MicroRNA dynamics in the stages of tumorigenesis correlate with hallmark capabilities of cancer. Genes & development.

[26] Iliopoulos D, Lindahl-Allen M, Polytarchou C, Hirsch HA, Tsichlis PN, Struhl K (2010). Loss of miR-200 inhibition of Suz12 leads to polycomb-mediated repression required for the formation and maintenance of cancer stem cells. Molecular cell.

[27] Shimono Y, Zabala M, Cho RW, Lobo N, Dalerba P, Qian D, Diehn M, Liu H, Panula SP, Chiao E, Dirbas FM, Somlo G, Pera RA, Lao K, Clarke MF (2009). Downregulation of miRNA-200c links breast cancer stem cells with normal stem cells. Cell.

[28] Adam L, Zhong M, Choi W, Qi W, Nicoloso M, Arora A, Calin G, Wang H, Siefker-Radtke A, McConkey D, Bar-Eli M, Dinney C (2009). miR-200 expression regulates epithelial-to-mesenchymal transition in bladder cancer cells and reverses resistance to epidermal growth factor receptor therapy. Clinical cancer research : an official journal of the American Association for Cancer Research.

[29] Bryant JL, Britson J, Balko JM, Willian M, Timmons R, Frolov A, Black EP (2012). A microRNA gene expression signature predicts response to erlotinib in epithelial cancer cell lines and targets EMT. British journal of cancer.

[30] Cochrane DR, Howe EN, Spoelstra NS, Richer JK (2010). Loss of miR-200c: A Marker of Aggressiveness and Chemoresistance in Female Reproductive Cancers. Journal of oncology.

[31] Cochrane DR, Spoelstra NS, Howe EN, Nordeen SK, Richer JK (2009). MicroRNA-200c mitigates invasiveness and restores sensitivity to microtubule-targeting chemotherapeutic agents. Molecular cancer therapeutics.

[32] Kopp F, Oak PS, Wagner E, Roidl A (2012). miR-200c sensitizes breast cancer cells to doxorubicin treatment by decreasing TrkB and Bmi1 expression. PloS one.

[33] Tryndyak VP, Beland FA, Pogribny IP (2010). E-cadherin transcriptional down-regulation by epigenetic and microRNA-200 family alterations is related to mesenchymal and drug-resistant phenotypes in human breast cancer cells. International journal of cancer Journal international du cancer.

[34] Lewis BP, Shih IH, Jones-Rhoades MW, Bartel DP, Burge CB (2003). Prediction of mammalian microRNA targets. Cell.

[35] John B, Enright AJ, Aravin A, Tuschl T, Sander C, Marks DS (2004). Human MicroRNA targets. PLoS biology.

[36] Maragkakis M, Alexiou P, Papadopoulos GL, Reczko M, Dalamagas T, Giannopoulos G, Goumas G, Koukis E, Kourtis K, Simossis VA, Sethupathy P, Vergoulis T, Koziris N, Sellis T, Tsanakas P, Hatzigeorgiou AG (2009). Accurate microRNA target prediction correlates with protein repression levels. BMC bioinformatics.

[37] Maragkakis M, Reczko M, Simossis VA, Alexiou P, Papadopoulos GL, Dalamagas T, Giannopoulos G, Goumas G, Koukis E, Kourtis K, Vergoulis T, Koziris N, Sellis T, Tsanakas P, Hatzigeorgiou AG (2009). DIANA-microT web server: elucidating microRNA functions through target prediction. Nucleic acids research.

[38] Lim LP, Lau NC, Garrett-Engele P, Grimson A, Schelter JM, Castle J, Bartel DP, Linsley PS, Johnson JM (2005). Microarray analysis shows that some microRNAs downregulate large numbers of target mRNAs. Nature.

[39] Hollestelle A, Elstrodt F, Nagel JH, Kallemeijn WW, Schutte M (2007). Phosphatidylinositol-3-OH kinase or RAS pathway mutations in human breast cancer cell lines. Molecular cancer research : MCR.

[40] Kozma SC, Bogaard ME, Buser K, Saurer SM, Bos JL, Groner B, Hynes NE (1987). The human c-Kirsten ras gene is activated by a novel mutation in codon 13 in the breast carcinoma cell line MDA-MB231. Nucleic acids research.

[41] Sunaga N, Shames DS, Girard L, Peyton M, Larsen JE, Imai H, Soh J, Sato M, Yanagitani N, Kaira K, Xie Y, Gazdar AF, Mori M, Minna JD (2011). Knockdown of oncogenic KRAS in non-small cell lung cancers suppresses tumor growth and sensitizes tumor cells to targeted therapy. Molecular cancer therapeutics.

[42] Tuveson DA, Shaw AT, Willis NA, Silver DP, Jackson EL, Chang S, Mercer KL, Grochow R, Hock H, Crowley D, Hingorani SR, Zaks T, King C, Jacobetz MA, Wang L, Bronson RT (2004). Endogenous oncogenic K-ras(G12D) stimulates proliferation and widespread neoplastic and developmental defects. Cancer cell.

[43] Wang XQ, Li H, Van Putten V, Winn RA, Heasley LE, Nemenoff RA (2009). Oncogenic K-Ras regulates proliferation and cell junctions in lung epithelial cells through induction of cyclooxygenase-2 and activation of metalloproteinase-9. Molecular biology of the cell.

[44] Agbunag C, Bar-Sagi D (2004). Oncogenic K-ras drives cell cycle progression and phenotypic conversion of primary pancreatic duct epithelial cells. Cancer research.

[45] Fan J, Bertino JR (1997). K-ras modulates the cell cycle via both positive and negative regulatory pathways. Oncogene.

[46] Shimizu K, Birnbaum D, Ruley MA, Fasano O, Suard Y, Edlund L, Taparowsky E, Goldfarb M, Wigler M (1983). Structure of the Ki-ras gene of the human lung carcinoma cell line Calu-1. Nature.

[47] Valenzuela DM, Groffen J (1986). Four human carcinoma cell lines with novel mutations in position 12 of c-K-ras oncogene. Nucleic acids research.

[48] She QB, Chandarlapaty S, Ye Q, Lobo J, Haskell KM, Leander KR, DeFeo-Jones D, Huber HE, Rosen N (2008). Breast tumor cells with PI3K mutation or HER2 amplification are selectively addicted to Akt signaling. PloS one.

[49] Uhlmann S, Zhang JD, Schwager A, Mannsperger H, Riazalhosseini Y, Burmester S, Ward A, Korf U, Wiemann S, Sahin O (2010). miR-200bc/429 cluster targets PLCgamma1 and differentially regulates proliferation and EGF-driven invasion than miR-200a/141 in breast cancer. Oncogene.

[50] Park IK, Morrison SJ, Clarke MF (2004). Bmi1, stem cells, and senescence regulation. The Journal of clinical investigation.

[51] Marusyk A, Almendro V, Polyak K (2012). Intra-tumour heterogeneity: a looking glass for cancer?. Nature reviews Cancer.

[52] Saunders NA, Simpson F, Thompson EW, Hill MM, Endo-Munoz L, Leggatt G, Minchin RF, Guminski A (2012). Role of intratumoural heterogeneity in cancer drug resistance: molecular and clinical perspectives. EMBO molecular medicine.

[53] Howe EN, Cochrane DR, Richer JK (2012). The miR-200 and miR-221/222 microRNA families: opposing effects on epithelial identity. Journal of mammary gland biology and neoplasia.

[54] Philipp A, Zhao X, Tarcha P, Wagner E, Zintchenko A (2009). Hydrophobically modified oligoethylenimines as highly efficient transfection agents for siRNA delivery. Bioconjugate chemistry.

[55] Rasband WS (1997).

[56] Chin LJ, Ratner E, Leng S, Zhai R, Nallur S, Babar I, Muller RU, Straka E, Su L, Burki EA, Crowell RE, Patel R, Kulkarni T, Homer R, Zelterman D, Kidd KK (2008). A SNP in a let-7 microRNA complementary site in the KRAS 3' untranslated region increases non-small cell lung cancer risk. Cancer research.

