# Supplementary material for: The proto-oncogene KRAS is targeted by miR-200c
Source: Oncotarget. 2013 Nov 24;5(1):185–95. doi: 10.18632/oncotarget.1427 (PMC3960200; doi:10.18632/oncotarget.1427)
Supplement: Supplementary file 1 [file oncotarget-05-0185-s001.pdf]

The proto-oncogene KRAS is targeted by miR-200c - Kopp et al

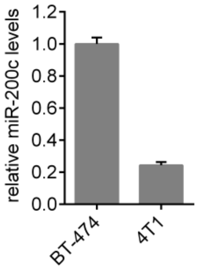

Supplement Figure S1: miR-200c expression in the murine breast cancer cell line 4T1. The relative miR-200c expression of 4T1 cells in comparison to BT-474 cells was determined by quantitative RT-PCR.

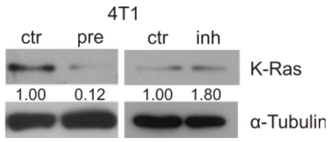

Supplement Figure S2: Regulation of K-ras protein expression in 4T1 cells upon miR-200c overexpression and inhibition. K-ras protein levels were examined by Western blot analysis at 72 hours after transfection with either pre-miR-200c (pre) (left panel) or miR-200c inhibitor (inh) (right panel). The relative intensities of the Western blots were quantified using ImageJ software. K-ras signals were then normalized to the loading control α-tubulin and controls (ctr) were set at 1.00.

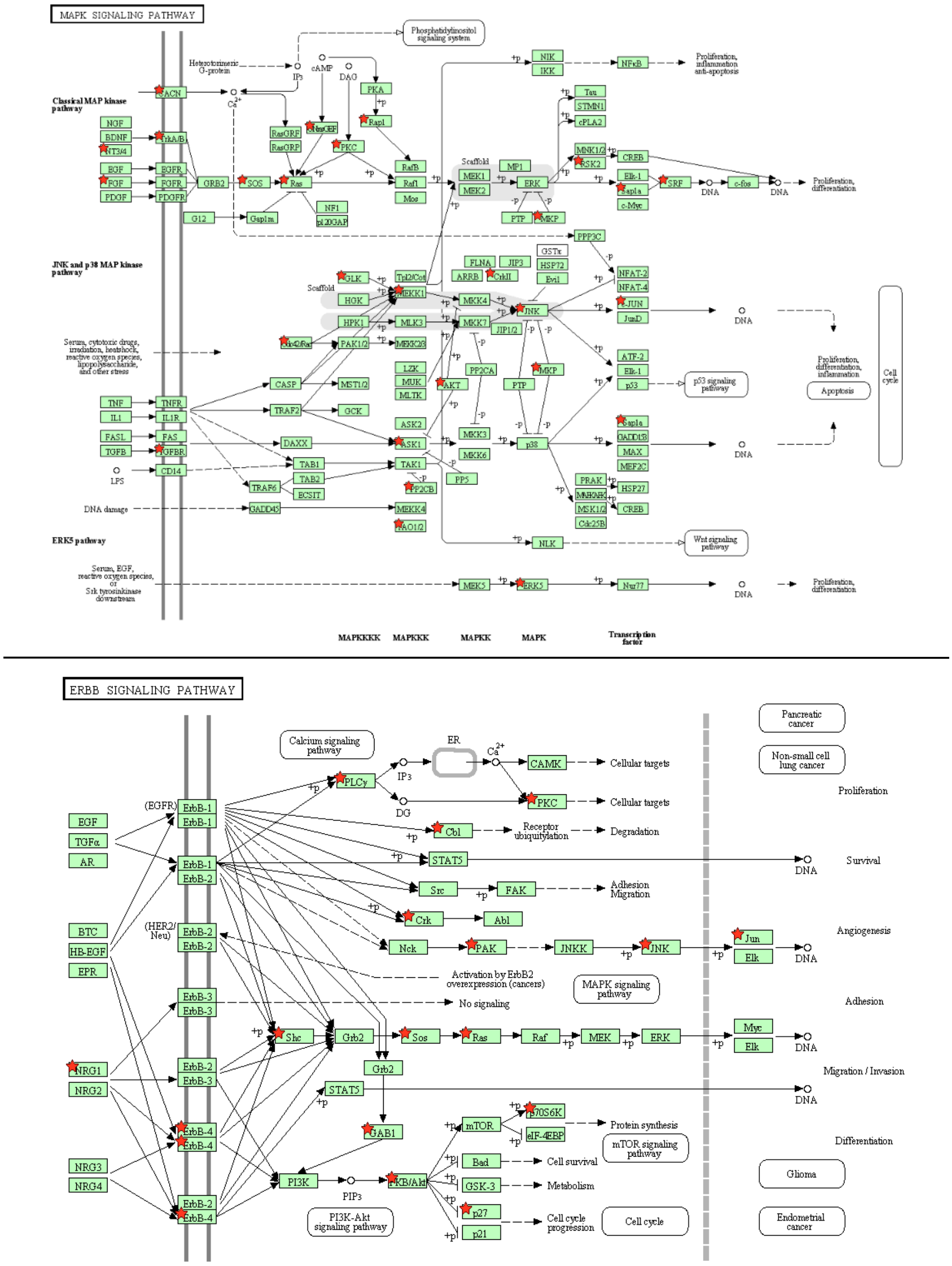

Supplement Figure S3: KEGG-pathway analysis of predicted miR-200c targets. An analysis of all potential targets of miR-200c predicted by TargetScan was carried out using the Database for Annotation, Visualization and Integrated Discovery (DAVID) and visualized by KEGG-pathway maps [1, 2]. Red asterisks represent predicted targets of miR-200c. (Upper panel: MAPK signaling pathway. Lower panel: ERBB signaling pathway)

REFERENCES

1. Huang da W, Sherman BT and Lempicki RA. Systematic and integrative analysis of large gene lists using DAVID bioinformatics resources. Nature protocols. 2009; 4(1):44-57.
2. Huang da W, Sherman BT and Lempicki RA. Bioinformatics enrichment tools: paths toward the comprehensive functional analysis of large gene lists. Nucleic acids research. 2009; 37(1):1-13.
