# Supplementary material for: The proto-oncogene KRAS is targeted by miR-200c
Source: Oncotarget. 2013 Nov 24;5(1):185–95. doi: 10.18632/oncotarget.1427 (PMC3960200; doi:10.18632/oncotarget.1427)
Supplement: Supplementary file 2 [file oncotarget-05-0185-s002.pdf]

Supplement Table S1: Primers and UPL hydrolysis probes (Roche) used for the quantitative RT-PCR. (F = forward primer; R = reverse primer; SLP = stem loop primer)

Primer (UPL probe)	Sequence (5’ – 3’)
KRAS F (#42)	TGGACGAATATGATCCAACAAT
KRAS R (#42)	TCCCTCATTGCACTGTACTCC
GAPDH F (#60)	AGCCACATCGCTCAGACAC
GAPDH R (#60)	GCCCAATACGACCAAATCC
miR-200c SLP	GTTGGCTCTGGTGCAGGGTCCGAGGTATTCGCACCAGAGCCAACTCCATC
miR-200c F	GCGTAATACTGCCGGGTAAT
miR-191 SLP	GTTGGCTCTGGTGCAGGGTCCGAGGTATTCGCACCAGAGCCAACCAGCTG
miR-191 F	GCGCAACGGAATCCCAAAAG
universal R	GTGCAGGGTCCGAGGT
